# Utilization of oleo-chemical industry by-products for biosurfactant production

**DOI:** 10.1186/2191-0855-3-68

**Published:** 2013-11-21

**Authors:** Garima Bhardwaj, Swaranjit Singh Cameotra, Harish Kumar Chopra

**Affiliations:** 1 Department of Chemistry, Sant Longowal Institute of Engineering and Technology, Longowal, 148106, Distt. Sangrur (Pb.), India; 2 Institute of Microbial Technology, Sector-39-A, Chandigarh 160036, India

**Keywords:** Biosurfactants, Agro- chemical waste, Rhamnolipid, Oil industry

## Abstract

Biosurfactants are the surface active compounds produced by micro-organisms. The eco-friendly and biodegradable nature of biosurfactants makes their usage more advantageous over chemical surfactants. Biosurfactants encompass the properties of dropping surface tension, stabilizing emulsions, promoting foaming and are usually non- toxic and biodegradable. Biosurfactants offer advantages over their synthetic counterparts in many applications ranging from environmental, food, and biomedical, cosmetic and pharmaceutical industries. The important environmental applications of biosurfactants include bioremediation and dispersion of oil spills, enhanced oil recovery and transfer of crude oil. The emphasis of present review shall be with reference to the commercial production, current developments and future perspectives of a variety of approaches of biosurfactant production from the micro-organisms isolated from various oil- contaminated sites and from the by-products of oleo-chemical industry wastes/ by-products viz. used edible oil, industrial residues, acid oil, deodorizer distillate, soap-stock etc.

## Introduction

Biosurfactants are the surface active agents that are amphipathic in nature and possess both hydrophilic and hydrophobic moieties that reduce the surface and interfacial tensions between two immiscible liquids. The polar and non-polar moieties present in the structure of biosurfactants allow them to accumulate at inter-phase between liquids of different polarities and form micelles thereby reducing surface tension and facilitating hydrocarbon uptake and emulsification. The interest in biosurfactants is taking much more attention these days due to their promising quality towards the environment. The biosurfactants are preferred over their chemically synthesised counterparts because of their higher biodegradability and selective nature towards the environmental factors like temperature, pH, and salinity. However, the biosurfactants are not able to compete with the chemical surfactants due to their higher production costs (Gautam and Tyagi 2006; Pacwa-Plociniczak et al. 2011). Their future completely depends upon the economic balance between their production costs, functional benefits and the development of economical processes by the use of low cost raw materials (Cameotra and Makkar 1998; Desai and Banat 1997). Therefore a lot of wastes are getting attention in response to reduce the cost of biosurfactant production and their use world-wide for a better environment. Some of these wastes include vegetable oils, distillery and dairy wastes which can be used efficiently to reduce the biosurfactant production costs (Makkar and Cameotra 2002). Oil industry waste in the form of by-products generated during the manufacture of vegetable oil is found to be very useful. Therefore our main purpose is to consider various oil industry wastes for the production of biosurfactants as efficient and economical raw materials in order to lower the cost of biosurfactant production.

**Figure 1:**
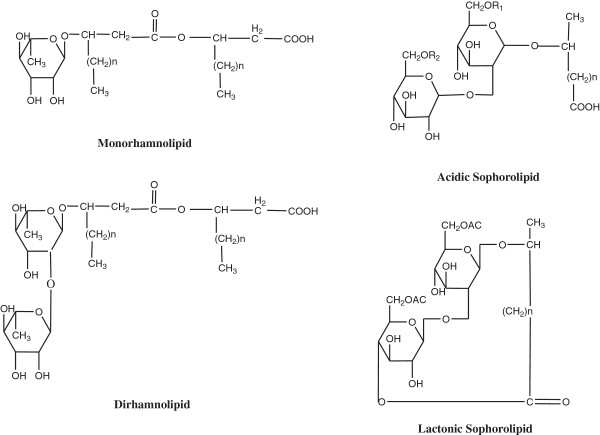
Various types of biosurfactants produced by microorganisms.

The process of biosurfactant production can be optimized by making the possible links between the production conditions, structure and function of these compounds (Makkar et al. 2011). Biosurfactants are widely used in hydrocarbon bioremediation field due to their enhanced oil recovery (EOR). Their presence lowers the surface and interfacial tension of the oil reservoirs which facilitate the oil flow and thus enhance the oil recovery (Kosaric 1992). Besides their use in enhanced oil recovery soil remediation is also of great importance where they accelerate the remediation of organic and metal contaminated sites (Christofi and Ivshina 2002). Other potential applications of biosurfactants relate to food, cosmetic, health care industries and cleaning toxic chemicals of industrial, agricultural origin and industrial waste utilization. The chemical structures of some of the common biosurfactants isolated from oleo-chemical industry waste are shown in Figure 1.

### Micro-organisms producing biosurfactants

A variety of micro-organisms produce biosurfactants that are diverse in chemical composition. The nature and amount of the biosurfactant produced solely depends upon the site from where the micro-organism is isolated and the various nutritional factors available for their growth (Table 1). Many microorganisms have been isolated from contaminated soils, effluents and waste water sources for industrial utilization of the various types of agro-industrial waste products. Thus, these have an ability to grow on substrates considered potentially noxious for other non-producing microorganisms.

**Table 1:** Potential biosurfactants with their producing micro-organisms

**Microorganism**	**Sources of isolation**	**By-products/ Carbon Sources**	**Biosurfactant**	**Reference**
*Pseudomonas sp.*	Oil spilled soil	Glucose/ Molasses/ Cheese whey	Rhamnolipid	Anandaraj and Thivakaran 2010
*Pseudomonas sp.*	Used edible oil	Used edible oil/ Rice-water/ Diesel/ Petrol/ Whey	Rhamnolipid	Soniyamby et al. 2011
*Bacillus subtilis*	Crude oil contaminated localities	Glucose/ Rapeseed oil supplemented with crude oil	Iturin	Bayoumi et al. 2010
*Bordetella hinizi-DAFI*	Crude oil contaminated localities	Sucrose/ Molasses supplemented with crude oil	Trehalose-2,3,4,2’-tetraester	Bayoumi et al. 2010
*Trichosporon asahii*	Petroleum-contaminated soil	Diesel oil	Sophorolipids	2010
*Pseudomonas aeruginosa LBI*	Petroleum contaminated soil	Soap-stock	Rhamnolipids	Benincasa et al. 2002
*Serratia marcescens*	Petroleum contaminated soil	Glycerol	Lipopeptide	Anyanwu et al. 2011
*Candida sp. SY-16*	Oil-containing soil sample	Soybean oil and glucose	Mannosylerythritol (Glycolipid)	Kim et al. 1999
*Pseudomonas aeruginosa SP4*	Petroleum contaminated soil	Palm oil	Rhamnolipid	Sarachat et al. 2010
*Rhodococcus sp.*	Oil-contaminated soil	Sucrose/ Kerosene/ n-heptane/ n-octane/ n-hexadecane/ n-paraffin/gas oil	Extra-cellular lipids and glycolipid	Shavandi et al. 2011
*Bacillus subtilis*	Oil contaminated soil	Vegetable oil/ Kerosene/ Petrol/ Diesel	Surfactin	Priya et al. 2009
*Pseudomonas aeruginosa*	Oil contaminated soil	Vegetable oil/ Kerosene/ Petrol/ Diesel	Rhamnolipid	Priya et al. 2009
*Pseudomonas aeruginosa* J4	Waste water of petrochemical factory	Glucose/ Diesel, Kerosene/Glycerol/ Olive Oil/ Sunflower oil/ Grape seed oil	Rhamnolipid	Wei et al. 2005
*Pseudomonas aeruginosa* EM1	Oil contaminated site	Glucose/ Glycerol/ Sucrose/ Hexane/ Olive oil/ Oleic acid/ soybean oil	Rhamnolipid	Wu et al. 2008

### Maximum production and recovery of biosurfactants via microbial bioprocess development

The bio-process optimization is a very important aspect of biosurfactant production using various industrial wastes. Several factors need to be considered before a standard procedure is laid out for the setting up the process at industrial level. These are described as follows.

#### Factors affecting the biosurfactant production

Many factors affect the production of biosurfactants at the genetic, nutritional and physicochemical environment levels. Several carbon and nitrogen sources which helps in the growth of micro-organisms but they are not suitable for the production of biosurfactants. Some of the carbon-nitrogen sources and the environmental factors affecting the production of biosurfactants selected from oil industry are discussed here.

#### Carbon sources

In case of hydrophilic substrates *Pichia anamola* supported better growth in glucose and in case of hydrophobic substrates soyabean and palm oil were the best carbon sources as compared to coconut oil as a very low amount of biosurfactant is produced on it. The surface tension reduced to 28mN/m suggesting the secretion of biosurfactants in the fermentation media (Thaniyavarn et al. 2008). When grown on used edible oil as a carbon source *Pseudomonas sp* was able to produce maximum yield of biosurfactant which was 7.6 g/L compared to rice water, diesel, petrol and whey (Soniyamby et al. 2011). Olive oil was the best carbon source for the production of biosurfactants by *Pseudomonas fluorescence* compared to the hexadecane and glucose which reduced the surface tension of the fermentation media to 38 dyne/cm and an emulsification activity of 49%. Hexadecane was also able to reduce the surface tension of the fermentation media but with only 10% emulsification activity while on glucose the strain grew without biosurfactant production (Abouseoud et al. 2007). Soybean oil and glucose were used as the carbon sources for the production of mannosylerythritol lipid from *Candida sp.* SY 16 which lowered the surface tension to 29 dyne/cm at critical micelle concentration of 10 mg/l (Kim et al. 1999). Biosurfactants produced from the Industrial residue by *Candida lipolytica* are very promising to their use in microbial enhanced oil recovery due to their high tolerance to NaCl concentrations which is mainly found in various oil reservoirs (Rufino et al. 2007). The optimized conditions for the production of biosurfactants from the *Pseudomonas aeruginosa* SCMU106 included a combination of glucose and corn oil (Techaoei et al. 2011). 4% soybean cooking oil was used as a carbon source for the production of monoacylglycerols by *Candida ishiwadae* strain isolated from plant material (Thanomsub et al. 2004). 2% of palm oil was used to obtain the highest concentrations of biosurfactants by *Pseudomonas aeruginosa* SP4 strain isolated from petroleum contaminated soil (Sarachat et al. 2010). When sunflower oil was used as the carbon source by *Tsukamurella spec.* DSM 44370 a mixture of oligosaccharide lipids were produced. In case the carbon source was replaced with calendula oil the nature of the biosurfactant changed (Langer et al. 2006). Biosurfactant production was enhanced during growth of *Nocardiopsis sp*. B4 when olive oil was used as the carbon source (Khopade et al. 2012). Three soybean oil refinement wastes; acid oil, deodorizer distillate and soapstock were used by the *Pseudomonas aeruginosa* MR01 as carbon sources to reduce the cost of biosurfactant production (Partovi et al. 2013). The use of soap-stock as the sole carbon source by *Pseudomonas aeruginosa* LBI resulted in the production of 16 g/L of the rhamnolipids (Benincasa et al. 2002). A strain *Pseudomonas aeruginosa* J4 isolated from the waste water of petrochemical industry was able to degrade the vegetable oil as well as mineral oil for the production of biosurfactants. The maximum production of biosurfactants was 3600 mg/L which was achieved with the 10% olive oil concentration (Wei et al. 2005).

#### Nitrogen sources

Yeast extract was the best nitrogen source used for the production of biosurfactant by *Bacillus* strains isolated from the marine sediments of Tamil Nadu coastal area. Also, the beef extract showed no significant change in biosurfactant production while used in place of yeast extract (Gnanamani et al. 2010). *Pseudomonas sp* showed the better yields of biosurfactant when grown on sodium nitrate as compared to the ammonia and urea (Soniyamby et al. 2011). *Pseudomonas fluorescence* growing on olive oil as the carbon source found to be more efficient biosurfactant producer with ammonium nitrate as the nitrogen source as compared to the sodium nitrate and ammonium chloride. Ammonium chloride was used for the growth but not for the biosurfactant production (Abouseoud et al. 2007). Peptone was found to be an essential component for the production of biosurfactants by *Lactobacillus paracasei ssp. Paracasei A20* while yeast extract was a promising component for the growth of bacteria. A combination of the peptone and meat extract showed an increase in the yield of biosurfactant compared to the standard media (Gudina et al. 2011). Phenylalanine was the most efficient nitrogen source for the cultivation of *Nocardiopsis sp.* B4 when used in combination with the olive oil as the carbon source (Khopade et al. 2012). The strain *Pseudomonas aeruginosa* EM1 isolated from the oil contaminated sites was screened for the use of various nitrogen sources to give the maximum production of biosurfactants and NaNO_3_ was found to be the best among NH_4_NO_3_, NH_4_Cl, urea and yeast extract (Wu et al. 2008).

#### Environmental factors affecting the production of biosurfactants

Growth conditions and environmental factors such as temperature, pH, salinity, agitation and oxygen availability also affect the production of biosurfactants. A lipopeptide biosurfactant produced by *Serratia marcescens* was able to retain its properties at high temperatures range up to 100°C, high NaCl concentrations up to 12% and a wide range of pH (Anyanwu et al. 2011). The optimum temperature and pH for the *Bacillus* strains isolated from the marine sediments of Tamil Nadu coastal area were 37°C and 7.2 ± 0.2 respectively (Gnanamani et al. 2010). The incubation time plays a significant role in the production of biosurfactants. The effect of incubation time can be seen by monitoring the values of emulsification activity, surface tension, biomass concentration after a regular interval of time. *Pseudomonas sp* showed the maximum rhamnolipid production of 5.86 g/L at 72 h (Soniyamby et al. 2011). *Pseudomonas fluorescence* after 36 h of incubation starts producing biosurfactant and reaches to its maximum concentration after about 56 h (Abouseoud et al. 2007). The product yield increased to 70% when aeration is supplied to the *Pseudomonas aeruginosa* LBI in a batch feed culture (Benincasa et al. 2002). In the batch fermentation of *Pseudomonas aeruginosa* EM1 when the agitation was increased from 50 to 250 rpm the rhamnolipid production increased to 80% (Wei et al. 2005).

### Applications

The biosurfactants possess a lot of applications ranging from environmental, food and biomedical, cosmetic and pharmaceutical industries. Some of the reported oil related applications are discussed here. The biosurfactants isolated from *Candida lipolytica, Candida antarctica, Candida bombicola, Torulopsis bombicola* and *Aspergillus ustus* were found to be the best choices in microbial enhanced oil recovery (Rufino et al. 2007; Kitamoto et al. 2001; Adamczak and Bednarski 2000; Felse et al. 2007; Cooper and Paddock 1984; Seghal Kiran et al. 2009). The biosurfactant produced by *Lactobacillus delbrueckii* when grown on peanut oil was used in the bioremediation processes and helped in biodegradation of crude oil in laboratory scale microcosm experiments (Thavasi et al. 2011). *Rhodococcus sp.* isolated from the Iranian oil contaminated soil was able to recover 65% of the trapped oil in a sand pack column which suggests its applications in the enhanced oil recovery (Shavandi et al. 2011). The biosurfactant produced from *Serratia marcescens* NSK1 was able to remove 60% of the engine oil and 51% of kerosene in a soil column study which suggest its various applications in microbial enhanced oil recovery (Anyanwu et al. 2011). The modified biosurfactant of *Tsukamurella sp* showed novel biological activities (Langer et al. 2006). Glycolipids from *Ustilago maydis* FBD 12 showed significant antimicrobial activities against *Salmonella enteric Var. Typhimurium* and *Staphyloccocus aureus* (Alejandro et al. 2011). The biosurfactant from the strain *Pseudomonas aeruginosa* EM1 isolated from the waste water of petrochemical industry was stated to be a good one to be used in the biodegradation processes (Wei et al. 2005). These all properties show their potential of usage at industrial level for a greener environment.

## Conclusions

This present review provides the basic scientific information on the production and applications of biosurfactants from the oleo-chemical industrial wastes that is required to exploit natural processes and develop methods to hasten these processes for economically viable production of biosurfactants by the usage of oil industry wastes. Regardless of the advantages of biosurfactant synthesis, its industrial use is still limited due to the high costs involved in the production process. The economics of biosurfactant production may be significantly impacted through the use of inexpensive carbon substrates. In this review, we have presented a thorough investigation of oleo chemical industry waste as carbon sources for biosurfactant production. Rapid advances in the last few years helped in the understanding of the process of biosurfactant fermentation/ production by many microorganisms.

### Future prospects

The biodegradable and low toxicity of biosurfactants makes them very promising for use in environmental sciences. The commercial success of biosurfactants is still limited owing to their high production costs. Optimized growth conditions using inexpensive renewable wastes and novel, efficient methods for isolation and purification of biosurfactants could make their production more economically feasible. Another important aspect regarding biological remediation technologies is the use of biosurfactant in the process on a large scale.

## Competing interests

The authors declare that they have no competing interest.

## Authors' contributions

All authors read and approved the final version of the manuscript.
